# Cost-Effectiveness of the Use of Adjuvanted Quadrivalent Seasonal Influenza Vaccine in Older Adults in Ireland

**DOI:** 10.3390/vaccines11050933

**Published:** 2023-05-03

**Authors:** Van Hung Nguyen, Mansoor Ashraf, Joaquin F. Mould-Quevedo

**Affiliations:** 1 VHN Consulting Inc., Montreal, QC H2V 3L8, Canada; 2 Seqirus UK, Maidenhead, Berkshire SL6 8AD, UK; 3 Seqirus USA Inc., Summit, NJ 07901, USA

**Keywords:** adjuvanted influenza vaccine, cost-effectiveness, older adults, Ireland

## Abstract

Background: Enhanced vaccines (e.g., containing adjuvants) have shown increased immunogenicity and effectiveness in older adults, who often respond sub-optimally to conventional influenza vaccines. In this study, we evaluated the cost-effectiveness of an inactivated, seasonal, MF59-adjuvanted quadrivalent influenza vaccine (aQIV) for use in adults ≥ 65 years in Ireland. Methods: A published dynamic influenza model incorporating social contact, population immunity, and epidemiological data was used to assess the cost-effectiveness of aQIV in adults ≥ 65 years of age compared with a non-adjuvanted QIV. Sensitivity analysis was performed for influenza incidence, relative vaccine effectiveness, excess mortality, and the impact on bed occupancy from co-circulating influenza and COVID-19. Results: The use of aQIV resulted in discounted incremental cost-effectiveness ratios (ICERs) of EUR 2420/quality-adjusted life years (QALYs) and EUR 12,970/QALY from societal and payer perspectives, respectively, both of which are below the cost-effectiveness threshold of EUR 45,000/QALY. Sensitivity analysis showed that aQIV was effective in most scenarios, except when relative vaccine effectiveness compared to QIV was below 3%, and resulted in a modest reduction in excess bed occupancy. Conclusion: The use of aQIV for adults ≥ 65 years old in Ireland was shown to be highly cost-effective from both payer and societal perspectives.

## 1. Introduction

Vaccination is the most effective available method of protection against seasonal influenza and its related complications. In Ireland, vaccination is offered free of charge through the National Immunisation Office for high-risk groups, which include older adults (≥65 years); pregnant women; healthcare workers; people with or living with/caring for people who have health conditions that put them at higher risk of influenza (e.g., long-term respiratory diseases, chronic heart conditions, or a weakened immune system); children aged 2 to 17 years; people living in long-term care facilities; and people with close regular contact with pigs, poultry, or waterfowl [1]. Despite a drop in vaccination rates during 2019–2020, vaccination coverage among adults aged 65 years and older has been steadily rising from 54.5% in the 2016–2017 season to 75.4% in the 2021–2022 season [2,3].

The effectiveness of seasonal influenza vaccines varies from season to season, depending at least in part on the degree of match between the vaccine and circulating strains [4]. This varying effectiveness across seasons has been shown to be consistent across age groups and regions, with vaccine effectiveness within any season decreasing with increasing age [5]. Older adults often have a poorer response to vaccines than younger adults, in part due to immunosenescence, the age-related decline in innate and adaptive immune functions [6]. This reduced capacity to effectively respond to influenza infection results in a high proportion of serious cases and deaths in this age group [7,8]. Moreover, older adults respond particularly poorly to the A/H3N2 component of the vaccine [4], the viral subtype that disproportionally affects adults aged 65 years and older [9]. Adjuvanted and high-dose influenza vaccines are now available to help overcome these challenges and increase effectiveness in older adults.

aQIV, an MF59^®^-adjuvanted quadrivalent influenza vaccine (aQIV), is recommended for use in older adults in many countries worldwide. Originally developed as a trivalent formulation, the vaccine has demonstrated significantly higher effectiveness compared with non-adjuvanted standard-dose influenza vaccines in older adults [10,11,12,13,14,15] and similar or higher effectiveness compared with high-dose quadrivalent influenza vaccines (QIVs) [11,12,16]. While recommended for use in adults 65 years and older in Ireland, aQIV was not available for the 2022–2023 season [1,17]. Analyses in other countries have shown that the use of aQIV in older adults is highly cost-effective in terms of reducing the burden of disease on healthcare services and increasing quality-of-life years (QALYs) gained, despite having higher vaccine costs compared with conventional standard-dose QIVs [18,19,20,21].

Since the start of the COVID-19 pandemic, additional burdens have been placed on healthcare services, with increased rates of hospitalisation and intensive care unit occupancy from infectious diseases compared with pre-pandemic years. The co-circulation of COVID-19 and influenza during the winter seasons has the potential to place additional pressure on healthcare services every year; therefore, the prevention of influenza to reduce the burden on hospital bed availability is especially important in older adults, who are particularly vulnerable to both diseases. In addition, the reduced circulation of influenza viruses related to social distancing measures during the COVID-19 pandemic has reduced population-level immunity [22], meaning that more people may be vulnerable to severe influenza outcomes and disease complications requiring hospital treatment compared with previous years. As vaccination remains the most important tool for disease prevention and reducing disease severity, the selection of the most effective vaccines for each age group is critical for reducing both the individual risk of disease and the burden on healthcare services. In line with the increased risk of severe diseases and complications of influenza in older adults, in this analysis, we evaluate the cost-effectiveness of aQIV in adults aged 65 years and older in Ireland compared with the current use of non-adjuvanted QIVs in this age group.

## 2. Methods

### 2.1. Model Design and Parameters

The model used in this analysis was based on that used by Baguelin et al. (2013) [23] and included an adapted dynamic Susceptible–Exposed–Infected–Recovered (SEIR) influenza model that incorporated sentinel data, as well as data from primary and secondary care sources, on epidemiology, social contact, and immunity to influenza viruses in the population (Supplementary Figure S1a). The outcomes of symptomatic infections were assumed to be either non-medically attended, requiring a general practitioner (GP) visit, or requiring hospitalisation (Supplementary Figure S1b). The full details of the model have been reported previously [23,24]. In brief, the model used was an age-structured, four-strain (strains A/H1N1, A/H3N2, B/Victoria, and B/Yamagata) dynamic model, where vaccination was modelled by moving individuals from the susceptible (S) component to the recovered (R) component. In line with a previous analysis, we assumed that 27% of the population started the season (pre-vaccination) protected from influenza infection (subclinical or clinical), and neither infection- nor vaccine-induced protection waned during the season [23].

In order to ensure the model validity, the probability of influenza transmission was calibrated to reflect the variability in influenza circulation over the year, using overall attack rates of 7%, 10%, and 14% based on data reported prior to the COVID-19 pandemic [25]. Attack rates were adjusted by age group based on the varying degrees of mixing estimated via the contact matrix. Based on a total population of Ireland of 5.012 million individuals [26], this reflected a scenario of “no vaccination”, resulting in 428,051 cases for the 7% attack rate, 600,524 cases for the 10% attack rate, and 669,778 cases for the 14% attack rate. The transmissibility parameter *q* was estimated using the Nelder–Mead simplex procedure and maximum likelihood assessment. UK-specific data from the POLYMOD study were used to estimate the contact matrix between the age groups [27].

In contrast to the original analysis by Baguelin et al., no primary epidemiological data were available; therefore, most of the parameters used in the model were taken directly from estimates of central values of posterior distributions calculated previously by Baguelin and colleagues [23]. Ireland-specific values were used as inputs where available; otherwise, parameters were taken from a recent UK-based analysis [20]. As in the UK analysis, data on hospitalisations during the 2017–2018 influenza season were used to estimate symptomatic cases across six age groups from 6 months to ≥75 years, with the probability of influenza-related GP visits, hospitalisations, and deaths calculated per group and risk category (Supplementary Table S1) [28]. Vaccine coverage parameters were based on data used for a similar analysis in the UK [20,29] and varied per age and risk group, with the highest uptake in the ≥75-year-old low-risk and high-risk groups (Table 1).

Vaccine effectiveness estimates by age were based on those used by Thorrington et al. (2019) [30] and were averaged across strains (Supplementary Table S2). The relative vaccine effectiveness (rVE) of aQIV vs. QIV was also estimated by age group (65–74 years and ≥75 years) (Table 2) based on the values used by Coleman et al. (2021) [15]. Based on this, rVE for the base case scenario was set at 13.9%.

### 2.2. Scenarios

The current influenza immunisation strategy (current vaccination scenario) was compared to a strategy where aQIV was used in adults ≥65 years of age (aQIV scenario) (Supplementary Figure S2). Both payer and societal perspectives were used for the analysis; the payer analysis included the costs of GP visits, hospitalisation, the vaccine, and vaccine administration, whereas the societal analysis also included the impact of the loss of productivity.

Two vaccines—a non-adjuvanted egg-based QIV and a quadrivalent (intranasally administered) live-attenuated influenza vaccine (QLAIV)—were included in the model, with the addition of aQIV for adults aged ≥65 years of age in the aQIV scenario only. For both scenarios, QLAIV was used for children aged 2–17 years, and QIV was used for at-risk patients 18–64 years old. In the current vaccination scenario, QIV was used for patients ≥ 65 years old, whereas in the aQIV scenario, patients ≥ 65 years old would receive aQIV.

### 2.3. Economic Evaluations

The economic input parameters were based on estimates used by Sandmann et al. (2022) [31]. GP costs were EUR 50 per event; hospitalisations were EUR 5000 per event, and the quality-adjusted life years (QALYs) associated with outpatient and inpatient visits were 0.0078 and 0.017, respectively. Vaccine prices were set as EUR 10 for QIV, EUR 18 for aQIV, and EUR 18 for QLAIV, based on current average market prices across Europe [32,33]. As workforce absence due to influenza can result in substantial indirect costs (i.e., loss of productivity), we estimated the impact of each scenario based on an average across age groups of 4 days of lost productivity due to a GP visit (low- and high-risk patients) and 11 days and 22 days lost for hospitalisation for low- and high-risk patients, respectively. The costs of lost productivity were based on the average wage in Ireland of EUR 51,676 per annum [34]. As the safety profiles of the vaccines are similar, potential adverse events leading to healthcare usage or absenteeism were not considered in the economic model. While short-term local reactogenicity is higher for aQIV than for the other vaccines, this was not considered to have a measurable impact on the economic outcomes and was excluded from the model for simplicity [35].

The cost-effectiveness of the vaccine was assessed in terms of discounted life years lost, QALYs, and incremental cost-effectiveness ratios (ICERs) against a cost-effectiveness threshold of EUR 45,000/QALY [36,37]. QALYs were discounted at an annual rate of 3%.

### 2.4. Sensitivity Analysis

As the results from the model are sensitive to input parameters, such as estimates of vaccine efficacy and effectiveness, vaccine cost, and disease incidence, we estimated the impacts of changes in these parameters within probable boundaries. A sensitivity analysis was performed, which included variations in influenza incidence, strain prevalence, aQIV rVE, and excess mortality rates due to the overburdening of healthcare services from the co-circulation of other diseases (e.g., COVID-19). In addition, the impact on hospital and intensive care unit (ICU) bed occupancy from co-circulating influenza and COVID-19 was also evaluated, assuming 14,412 total available hospital beds [38], 350 ICU beds [39], and an 85% occupancy rate for both hospital and ICU beds [40]. Other parameters regarding bed occupancy were the same as those published previously [41]. For strain prevalence, the predominant strain was assumed to be associated with 70% of cases, and the other 30% were split across the remaining strains. For aQIV rVE, values were varied from 3% to 24%. For excess mortality caused by the overburdening of healthcare services (i.e., when ICU bed availability is exceeded), the impact of aQIV on influenza-related deaths, QALYs, and ICERs was estimated for 1.25-, 1.5-, 1.75-, and 2-fold death rates over the base case scenarios.

### 2.5. Software

The model was developed using R software and C++, as outlined by Nguyen et al. (2022) [41], predominantly using the following packages and corresponding libraries: Rcpp 1.0.9, RcppArmadillo 0.11.2.3.1, and RcppGSL 0.3.11. Model calibration was performed using the nloptr package.

## 3. Results

The use of aQIV in adults ≥ 65 years old would result in modest reductions in GP visits, hospital visits, and deaths (Table 3). Overall, switching from QIV to aQIV in this age group would reduce the annual number of symptomatic cases by 4107, GP visits by 411, hospitalisations by 156, and deaths by 42. This equates to a reduction in total QALYs lost of 275 compared with the current vaccination scenario.

Overall, the aQIV scenario resulted in cost savings compared with the current vaccination scenario in terms of GP costs, hospitalisation costs, and costs associated with the loss of productivity (Table 3). However, due to the increased costs of the vaccine compared with QIV (EUR 18 vs. EUR 10/dose), the overall costs would be EUR 665,240 higher compared with the current vaccination scenario. From a payer perspective, and based on 275 discounted QALYs gained, the ICER for the aQIV scenario was EUR 12,970/QALY, substantially below the EUR 45,000 threshold (Table 4). When the loss of productivity was also taken into account, the ICER from the societal perspective was EUR 2420/QALY. The cost-neutral price for aQIV would therefore be EUR 16.85 from a societal perspective and EUR 11.50 from a payer perspective.

**Figure 1:**
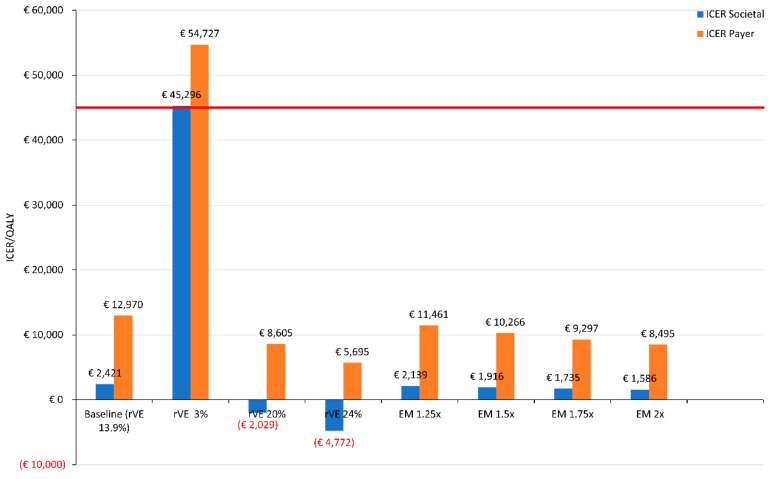
Sensitivity analysis of incremental cost-effectiveness ratios (ICERs) of using aQIV in adults ≥ 65 years over the baseline scenario (current vaccination scenario). Relative vaccine effectiveness (rVE) was varied from the baseline scenario of 13.9% to a range from 3% to 24%. Excess mortality above that expected from influenza alone was varied from 1.25-fold up to 2-fold. EM, excess mortality (fold increase); ICER, incremental cost-effectiveness ratio; QALY, quality-adjusted life year; rVE, relative vaccine effectiveness; ICER societal perspective includes costs associated with general practitioner visits, hospitalisation, loss of productivity, vaccine, and administration; ICER payer perspective includes costs associated with general practitioner visits, hospitalisation, vaccine, and administration.

**Figure 2:**
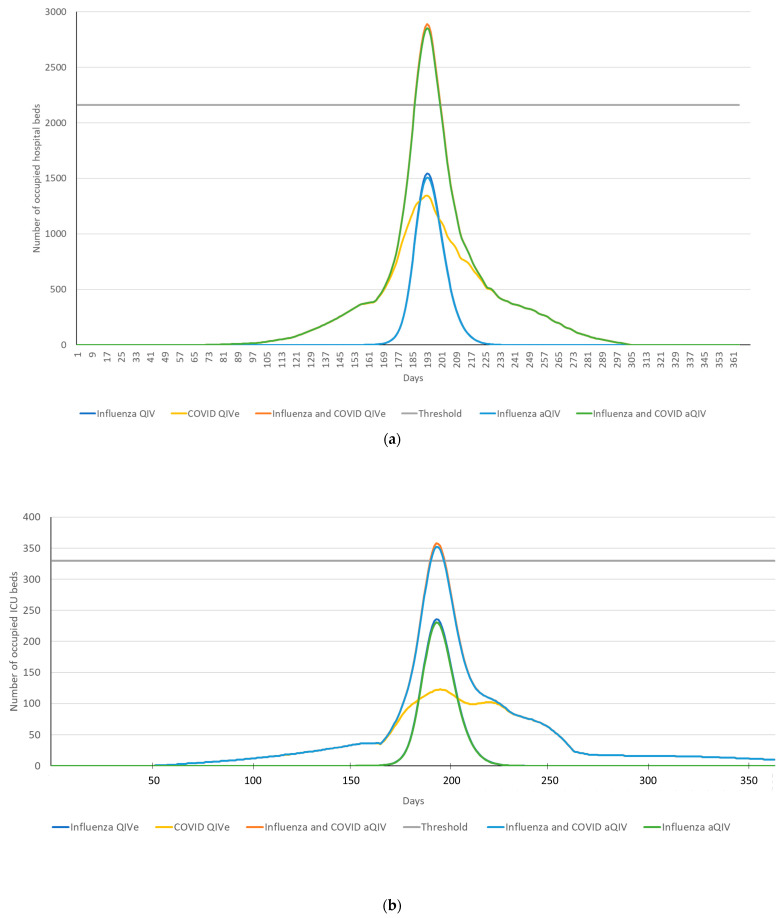
(**a**) Acute and (**b**) ICU bed occupancy estimates over time for the current vaccination (QIVe) and aQIV scenarios. Data are presented for number of beds occupied by patients with influenza only, COVID-19 only, and influenza or COVID-19 for the current vaccination (QIVe) and aQIV scenarios. “Acute” refers to hospital beds outside of the ICU. Estimates assume that peak bed occupancy occurs at the same time for both diseases. aQIV, adjuvanted quadrivalent influenza vaccine; ICU, intensive care unit; QIVe, egg-based quadrivalent influenza vaccine.

Sensitivity analysis showed that the use of aQIV in adults ≥65 years of age was cost-effective in most scenarios, except when rVE for aQIV vs. QIV dropped to 3% or below (Figure 1). Excess mortality above that expected from influenza alone (e.g., due to co-circulation of SARS-CoV-2) further increased the benefits of aQIV over QIV, with lower ICER with increasing excess mortality. From a payer perspective, ICER was reduced from EUR 11,461/QALY for 1.25-fold excess mortality to EUR 8495/QALY for 2-fold excess mortality. From a societal perspective, which included the additional impacts of the loss of productivity due to sickness, ICER dropped from EUR 2139/QALY to EUR 1586/QALY. Overall, the use of aQIV had a modest impact on total hospital and ICU bed occupancy from COVID-19 and influenza (Figure 2). Compared with the current vaccination scenario, the use of aQIV in older adults was estimated to result in 34 fewer hospital beds occupied and 5 fewer ICU beds (Table 5).

## 4. Discussion

In this study, the use of an MF59-adjuvanted quadrivalent influenza vaccine (aQIV) in older adults (≥65 years old) in Ireland was shown to be cost-effective, with an ICER of EUR 2420–EUR 12,970, when clinical and public health benefits were inputted into a published dynamic influenza transmission model. This ICER estimate was robust to sensitivity analysis for the influenza influence and strain prevalence, with all scenarios evaluated with an rVE > 3% for aQIV vs. QIV, resulting in ICERs considerably below the EUR 45,000 threshold recommended by the Government of Ireland [37]. The use of aQIV in older adults was also shown to result in a modest reduction in excess bed occupancy against a background of co-circulating COVID-19 and influenza.

To our knowledge, this is the first evaluation of the cost-effectiveness of an adjuvanted QIV for older adults in Ireland. Previous studies in other countries have also shown aQIV to be more cost-effective for use in older adults compared with standard-dose QIVs [18,19,21,28,42]. While the degree of cost-effectiveness varied with the vaccine price, cost benefits were mainly driven by the higher vaccine effectiveness of aQIV in this age group. As older adults are at the highest risk for influenza-related complications and mortality [43], the use of vaccines with higher effectiveness can have a substantial impact on healthcare utilisation. In light of this, a sensitivity analysis evaluating the effect of co-circulating SARS-CoV-2 and influenza on hospital bed occupancy showed a small but positive impact of aQIV usage in older adults on reductions in both non-ICU and ICU bed occupancy. These findings mirror those seen in similar analyses in the US and UK, where the use of aQIV in adults ≥ 65 years of age resulted in reduced influenza-related bed occupancy, which could potentially be critical in periods of peak viral circulation and hospital resource utilisation [20,41]. In addition, the potential benefits of the use of a more effective vaccine in older adults could also extend to the rest of the susceptible population by increasing population-level immunity and aiding in the protection of other high-risk groups [44]. This could also impact hospital resource utilisation, together with reduced absenteeism of healthcare staff from sickness or caring for sick dependents.

This study used a dynamic SEIR model to simulate the epidemiology of influenza and the effects of vaccination. We feel that this was the most appropriate model for this analysis, as it captures the natural infection process as well as the impact of vaccination on reducing the pool of susceptible individuals. While more complex models could have been used, these would have been limited by the availability of data for robustly estimating parameters. These SEIR models have been used effectively for the cost-effectiveness analysis of influenza vaccines in other situations, thereby making the results of this study comparable with those performed in other countries [20,41]. However, as with all simulations, this study has a number of limitations. Firstly, Ireland-specific data were not available for all the assumptions of the model parameters; therefore, the parameters used by Baguelin et al. (2013) [23] were used when specific parameters were not available. While these were considered the closest evidence available, additional sensitivity analyses were performed to adjust for potential differences between these assumptions and the situation in Ireland. In addition, for simplicity, a fixed value was used to estimate the total available hospital and ICU beds, whereas, in reality, this may vary regionally and annually and may be adaptable within the healthcare system. A second limitation was that our modelling approach was based on the entire country, whereas the circulation of SARS-CoV-2 and influenza, together with the availability and utilisation of healthcare resources, may vary across regions within Ireland at any given time. However, it is possible that disease peaks may vary regionally, allowing the transfer of patients within the country to areas with lower bed occupancy, thereby mitigating any variation in regional bed availability. For simplicity, the current model assumes that peak bed occupancy from COVID-19 and influenza occurs simultaneously, which may not always be the case and would alter the estimates of absolute bed occupancy and whether these would exceed threshold numbers of bed availability. Thirdly, the current model does not consider the association between mortality and access to care and assumes that all deaths from influenza occur in a hospital setting. Finally, circulating strains and influenza vaccine effectiveness vary annually; while we have tried to accommodate this variation using three different influenza attack rates for simplicity, this does not represent the full potential variation in susceptibility to infection. In particular, social distancing measures introduced during the COVID-19 pandemic dramatically changed the epidemiology of respiratory viruses, including influenza, with substantially reduced circulation of influenza viruses worldwide during the 2020–2021 and 2021–2022 seasons [25,45,46]. These low levels of viral circulation have potentially led to an immunity deficit, with the lack of exposure resulting in lower population-level immunity to these viruses [22]. In the 2022–2023 season, detection rates in Ireland appear to be similar to pre-pandemic levels, with a slightly earlier peak compared with previous years [47]. However, hospitalisation and ICU admission rates due to influenza appear to be higher than in recent pre-pandemic seasons, particularly in older adults, and mortality rates in the 2022–2023 season are so far more than double those reported in the 2018–2019 season [25,47]. Therefore, it remains of critical importance that this age group is offered the most suitable vaccine to reduce the burden of influenza and the potential for severe disease outcomes.

## 5. Conclusions

In summary, the use of an MF59-adjuvanted QIV in adults aged ≥65 years can increase vaccine effectiveness, reduce mortality, and reduce disease-related costs, such as hospitalisations and GP visits. The use of aQIV in older adults, instead of the current non-adjuvanted vaccine, was associated with an ICER of EUR 2420–12,970, which is far below the guideline threshold for Ireland of EUR 45,000. In addition, the use of aQIV has the potential for further reducing costs by helping to decrease the burden of co-circulating influenza and COVID-19 on hospital services, reducing both acute hospital and ICU admissions. Based on the results of this model, the use of aQIV for adults ≥ 65 years old can be considered cost-effective for implementation in Ireland.

## Figures and Tables

**Table 1:** Vaccine coverage rates by age group and scenario for low-risk and high-risk groups.

	Current Vaccination Scenario		aQIV Scenario	
Age Group	Low Risk	High Risk	Low Risk	High Risk
6–23 months	0	3.10%	0	3.10%
2–17 years	27.60%	48.6%	27.60%	48.6%
18–49 years	-	48.6%	-	48.6%
50–64 years	-	48.6%	40%	48.6%
65–74 years	68%	68%	68%	68%
≥75 years	80%	80%	80%	80%

**Table 2:** Vaccine effectiveness by age group for the three vaccines included in the analysis. Based on current vaccination recommendations, QIVe was assumed to be available for all age groups, aQIV was assumed to be available for adults ≥ 65 years, and QLAIV was assumed to be available for children aged 2–17 years.

Age Group	QIV ^a^	aQIV	QLAIV
6–23 months	62.5%	-	-
2–17 years	62.5%	-	62.5%
18–49 years	54.0%	-	-
50–64 years	54.0%	-	-
65–74 years	47.8%	55.0%	-
≥75 years	45.3%	52.9%	-

aQIV, adjuvanted quadrivalent influenza vaccine; QIV, quadrivalent influenza vaccine; QLAIV, quadrivalent live-attenuated influenza vaccine. ^a^ Based on estimates in Supplementary Table S2, assuming equal strain circulation.

**Table 3:** Effects of baseline (current vaccination scenario) and aQIV scenario on influenza outcomes, healthcare system usage, quality-adjusted life years lost, and economic costs. All economic values are given in euros.

	Current Vaccination Scenario	aQIV Scenario	Difference: aQIV−Current Vaccination Scenario
Symptomatic cases	492,790	488,683	−4107
GP visits	49,279	48,868	−411
Hospital visits	6812	6656	−156
Deaths	1183	1141	−42
Life years lost	13,271	12,949	−322
QALYs lost from death	10,096	9851	−245
QALYs lost from sickness	3132	3102	−30
Total QALYs lost	13,228	12,953	−275
GP costs (EUR)	2,463,948	2,443,417	−20,531
Hospitalisation costs (EUR)	34,061,699	33,278,426	−783,273
Loss of productivity costs (EUR)	323,786,375	320,887,491	−2,898,884
Vaccine costs (EUR)	12,859,341	17,227,268	+4,367,927
Vaccine administration costs (EUR)	25,908,065	25,908,065	0
Total costs (EUR)	399,079,428	399,744,668	+665,240

aQIV, adjuvanted quadrivalent influenza vaccine; GP, general practitioner; QALY, quality-adjusted life year.

**Table 4:** Incremental cost-effectiveness ratios (ICERs) for aQIV scenario compared with current vaccination scenario.

	Cost (EUR)	QALYs Gained (Discounted)	ICER
Societal perspective ^a^	665,240	275	2420
Payer perspective ^b^	3,564,123	275	12,970

ICER, incremental cost-effectiveness ratio; QALY, quality-adjusted life year; ^a^ cost estimates include general practitioner, hospital, loss of productivity, vaccine, and administration costs; ^b^ cost estimates include general practitioner, hospital, vaccine, and administration costs.

**Table 5:** Excess acute and ICU bed occupancy for patients with COVID-19 or influenza for the current vaccination scenario and use of aQIV in adults ≥ 65 years of age. “Excess” hospital beds prevented refers to the use of aQIV compared with the baseline scenario (current vaccination scenario).

Scenario	Hospital Beds Used	ICU Beds Used	Excess Hospital Beds Used	Excess ICU Beds Used	Excess Hospital Beds Prevented	Excess ICU Beds Prevented
Baseline ^a^	2713	353	552	23	Ref	Ref
aQIV scenario	2680	348	518	18	34	5

aQIV, adjuvanted quadrivalent vaccine; ICU, intensive care unit; Ref, reference. ^a^ Baseline refers to the current vaccination scenario.

## Data Availability

Data are available from the authors upon reasonable request.

## Supplementary Materials

The following supporting information can be downloaded at https://www.mdpi.com/article/10.3390/vaccines11050933/s1. Figure S1: (a) Susceptible–Exposed–Infected–Recovered influenza transmission model structure and (b) probability tree of outcomes from symptomatic infected; Figure S2: Model scenarios; Table S1: Assumptions of probabilities of events per influenza case in low- and high-risk groups [23]; Table S2: Vaccine effectiveness estimates by strain and age group [25].

## References

[1] HSE Immunisation Guidelines: Influenza. https://www.hse.ie/eng/health/immunisation/hcpinfo/guidelines/.

[2] HSE Seasonal Influenza Vaccine Uptake in those Attending GP Clinics and Pharmacies for Vaccination, Ireland 1 September 2021 to 17 July 2022. https://www.hpsc.ie/a-z/respiratory/influenza/seasonalinfluenza/surveillance/influenzaandadults65yearsandolder/Seasonal%20Flu%20Vacc%20Uptake_report_01%2009%202021%20-%20%2028%2007%202029_v1.0-%20final.pdf.

[3] HSE Seasonal Influenza Vaccine Uptake in Ireland in Persons Aged 65 Years and Older Attending GP Clinics and Pharmacies for Vaccination. https://www.hpsc.ie/a-z/respiratory/influenza/seasonalinfluenza/surveillance/influenzaandadults65yearsandolder/Seasonal%20Flu%20Vaccination%20Uptake_65%20report_Sep18-Aug%2019.docx.pdf.

[4] Belongia E.A., Simpson M.D., King J.P., Sundaram M.E., Kelley N.S., Osterholm M.T., McLean H.Q. (2016). Variable influenza vaccine effectiveness by subtype: A systematic review and meta-analysis of test-negative design studies. Lancet Infect. Dis..

[5] Okoli G.N., Racovitan F., Abdulwahid T., Righolt C.H., Mahmud S.M. (2021). Variable seasonal influenza vaccine effectiveness across geographical regions, age groups and levels of vaccine antigenic similarity with circulating virus strains: A systematic review and meta-analysis of the evidence from test-negative design studies after the 2009/10 influenza pandemic. Vaccine.

[6] Crooke S.N., Ovsyannikova I.G., Poland G.A., Kennedy R.B. (2019). Immunosenescence and human vaccine immune responses. Immun Ageing.

[7] Paget J., Spreeuwenberg P., Charu V., Taylor R.J., Iuliano A.D., Bresee J., Simonsen L., Viboud C., Global Seasonal Influenza-Associated Mortality Collaborator Network and GLaMOR Collaborating Teams (2019). Global mortality associated with seasonal influenza epidemics: New burden estimates and predictors from the GLaMOR Project. J Glob. Health.

[8] Nichol K.L. (2005). Influenza vaccination in the elderly: Impact on hospitalisation and mortality. Drugs Aging.

[9] Jester B.J., Uyeki T.M., Jernigan D.B. (2020). Fifty Years of Influenza A(H3N2) Following the Pandemic of 1968. Am. J. Public Health.

[10] Izurieta H.S., Chillarige Y., Kelman J., Wei Y., Lu Y., Xu W., Lu M., Pratt D., Wernecke M., MaCurdy T. (2020). Relative Effectiveness of Influenza Vaccines Among the United States Elderly, 2018–2019. J. Infect Dis..

[11] Boikos C., Fischer L., O’Brien D., Vasey J., Sylvester G.C., Mansi J.A. (2021). Relative Effectiveness of Adjuvanted Trivalent Inactivated Influenza Vaccine Versus Egg-derived Quadrivalent Inactivated Influenza Vaccines and High-dose Trivalent Influenza Vaccine in Preventing Influenza-related Medical Encounters in US Adults ≥ 65 Years During the 2017–2018 and 2018–2019 Influenza Seasons. Clin. Infect. Dis..

[12] Pelton S.I., Divino V., Shah D., Mould-Quevedo J., DeKoven M., Krishnarajah G., Postma M.J. (2020). Evaluating the Relative Vaccine Effectiveness of Adjuvanted Trivalent Influenza Vaccine Compared to High-Dose Trivalent and Other Egg-Based Influenza Vaccines among Older Adults in the US during the 2017-2018 Influenza Season. Vaccines.

[13] McConeghy K.W., Davidson H.E., Canaday D.H., Han L., Saade E., Mor V., Gravenstein S. (2021). Cluster-randomized Trial of Adjuvanted Versus Nonadjuvanted Trivalent Influenza Vaccine in 823 US Nursing Homes. Clin. Infect. Dis..

[14] Cocchio S., Gallo T., Del Zotto S., Clagnan E., Iob A., Furlan P., Fonzo M., Bertoncello C., Baldo V. (2020). Preventing the Risk of Hospitalization for Respiratory Complications of Influenza among the Elderly: Is There a Better Influenza Vaccination Strategy? A Retrospective Population Study. Vaccines.

[15] Coleman B.L., Sanderson R., Haag M.D.M., McGovern I. (2021). Effectiveness of the MF59-adjuvanted trivalent or quadrivalent seasonal influenza vaccine among adults 65 years of age or older, a systematic review and meta-analysis. Influenza Other Respir. Viruses.

[16] Izurieta H.S., Lu M., Kelman J., Lu Y., Lindaas A., Loc J., Pratt D., Wei Y., Chillarige Y., Wernecke M. (2021). Comparative Effectiveness of Influenza Vaccines Among US Medicare Beneficiaries Ages 65 Years and Older During the 2019–2020 Season. Clin. Infect. Dis..

[17] HSE Flu Vaccine—Overview. https://www2.hse.ie/conditions/flu/flu-vaccine/.

[18] Fochesato A., Sottile S., Pugliese A., Márquez-Peláez S., Toro-Diaz H., Gani R., Alvarez P., Ruiz-Aragón J. (2022). An Economic Evaluation of the Adjuvanted Quadrivalent Influenza Vaccine Compared with Standard-Dose Quadrivalent Influenza Vaccine in the Spanish Older Adult Population. Vaccines.

[19] Ruiz-Aragón J., Márquez-Peláez S., Gani R., Alvarez P., Guerrero-Luduena R. (2022). Cost-Effectiveness and Burden of Disease for Adjuvanted Quadrivalent Influenza Vaccines Compared to High-Dose Quadrivalent Influenza Vaccines in Elderly Patients in Spain. Vaccines.

[20] Nguyen V.H., Ashraf M., Mould-Quevedo J. (2023). Estimating the impact of influenza vaccination of low-risk 50–64-year-olds on acute and ICU hospital bed usage in an influenza season under endemic COVID-19 in the UK. Hum. Vaccin. Immunother..

[21] Calabrò G.E., Boccalini S., Panatto D., Rizzo C., Di Pietro M.L., Abreha F.M., Ajelli M., Amicizia D., Bechini A., Giacchetta I. (2022). The New Quadrivalent Adjuvanted Influenza Vaccine for the Italian Elderly: A Health Technology Assessment. Int. J. Environ. Res. Public Health.

[22] Dhanasekaran V., Sullivan S., Edwards K.M., Xie R., Khvorov A., Valkenburg S.A., Cowling B.J., Barr I.G. (2022). Human seasonal influenza under COVID-19 and the potential consequences of influenza lineage elimination. Nat. Commun..

[23] Baguelin M., Flasche S., Camacho A., Demiris N., Miller E., Edmunds W.J. (2013). Assessing optimal target populations for influenza vaccination programmes: An evidence synthesis and modelling study. PLoS Med..

[24] Baguelin M., Camacho A., Flasche S., Edmunds W.J. (2015). Extending the elderly- and risk-group programme of vaccination against seasonal influenza in England and Wales: A cost-effectiveness study. BMC Med..

[25] HSE Previous Influenza Seasons’ Surveillance Reports. https://www.hpsc.ie/a-z/respiratory/influenza/seasonalinfluenza/surveillance/influenzasurveillancereports/previousinfluenzaseasonssurveillancereports/.

[26] Central Statistics Office Popuilation and Migration Estimates, April 2021. https://www.cso.ie/en/releasesandpublications/ep/p-pme/populationandmigrationestimatesapril2021/mainresults/.

[27] Mossong J., Hens N., Jit M., Beutels P., Auranen K., Mikolajczyk R., Massari M., Salmaso S., Tomba G.S., Wallinga J. (2008). Social contacts and mixing patterns relevant to the spread of infectious diseases. PLoS Med..

[28] Kohli M.A., Maschio M., Mould-Quevedo J.F., Drummond M., Weinstein M.C. (2021). The cost-effectiveness of an adjuvanted quadrivalent influenza vaccine in the United Kingdom. Hum. Vaccin. Immunother..

[29] Public Health England Surveillance of Influenza and other Respiratory Viruses in the UK—Winter 2019 to 2020. https://webarchive.nationalarchives.gov.uk/ukgwa/20220401215804mp_/https://assets.publishing.service.gov.uk/government/uploads/system/uploads/attachment_data/file/895233/Surveillance_Influenza_and_other_respiratory_viruses_in_the_UK_2019_to_2020_FINAL.pdf.

[30] Thorrington D., van Leeuwen E., Ramsay M., Pebody R., Baguelin M. (2019). Assessing optimal use of the standard dose adjuvanted trivalent seasonal influenza vaccine in the elderly. Vaccine.

[31] Sandmann F.G., van Leeuwen E., Bernard-Stoecklin S., Casado I., Castilla J., Domegan L., Gherasim A., Hooiveld M., Kislaya I., Larrauri A. (2022). Health and economic impact of seasonal influenza mass vaccination strategies in European settings: A mathematical modelling and cost-effectiveness analysis. Vaccine.

[32] Influenza Vaccines 2022/2023. https://www.kvsa.de/fileadmin/user_upload/Bilder/Content/Praxis/Verordnung/22_02_18_Preisinformation_Grippeimpfstoffe_2022-2023_Tabelle_Homepage.pdf.

[33] National Institute for Health and Care Excellence (NICE) Medicinal Forms—Influenza Vaccines. https://bnf.nice.org.uk/drugs/influenza-vaccine/medicinal-forms/.

[34] Statista Average Annual Wage in Ireland from 2000 to 2021. https://www.statista.com/statistics/416212/average-annual-wages-ireland-y-on-y-in-euros/.

[35] Essink B., Fierro C., Rosen J., Figueroa A.L., Zhang B., Verhoeven C., Edelman J., Smolenov I. (2020). Immunogenicity and safety of MF59-adjuvanted quadrivalent influenza vaccine versus standard and alternate B strain MF59-adjuvanted trivalent influenza vaccines in older adults. Vaccine.

[36] O’Mahony J.F. (2021). Revision of Ireland’s Cost-Effectiveness Threshold: New State-Industry Drug Pricing Deal Should Adequately Reflect Opportunity Costs. PharmacoEconomics-Open.

[37] Government of Ireland Spending Review 2021: Review of High-Tech Drug Expenditure. https://assets.gov.ie/193851/9490e808-1774-440d-843a-28c3a9dc195c.pdf.

[38] Statista Number of Hospital Beds in Ireland from 2000 to 2020. https://www.statista.com/statistics/557287/hospital-beds-in-ireland/.

[39] HSE National Adult Critical Care Capacity—Census Report 2020. https://www.hse.ie/eng/about/who/cspd/ncps/critical-care/national-adult-critical-care-capacity-census-2020.pdf.

[40] OECD Hospital Beds and Occupancy. https://www.oecd-ilibrary.org/sites/e5a80353-en/index.html?itemId=/content/component/e5a80353-en.

[41] Nguyen V.H., Mould-Quevedo J.F. (2022). Estimating the Impact of Influenza Vaccination on Acute and ICU Hospital Bed Usage in an Influenza Season under Endemic COVID-19 in the US. Vaccines.

[42] Kohli M.A., Maschio M., Cartier S., Mould-Quevedo J., Fricke F.U. (2022). The Cost-Effectiveness of Vaccination of Older Adults with an MF59-Adjuvanted Quadrivalent Influenza Vaccine Compared to Other Available Quadrivalent Vaccines in Germany. Vaccines.

[43] Centers for Disease Control and Prevention Preliminary Estimated Influenza Illnesses, Medical visits, Hospitalizations, and Deaths in the United States—2021–2022 Influenza Season. https://www.cdc.gov/flu/about/burden/2021-2022.htm.

[44] Mertz D., Fadel S.A., Lam P.P., Tran D., Srigley J.A., Asner S.A. (2016). Herd effect from influenza vaccination in non-healthcare settings: A systematic review of randomised controlled trials and observational studies. Euro Surveill..

[45] European Centre for Disease Prevention and Control Seasonal Influenza 2021−2022—Annual Epidemiological Report. https://www.ecdc.europa.eu/en/publications-data/seasonal-influenza-2021-2022-annual-epidemiological-report.

[46] Lee S.S., Viboud C., Petersen E. (2022). Understanding the rebound of influenza in the post COVID-19 pandemic period holds important clues for epidemiology and control. Int. J. Infect. Dis..

[47] Health Protection Surveillance Centre Influenza Season 2022—2023. https://www.hpsc.ie/a-z/respiratory/influenza/seasonalinfluenza/surveillance/influenzasurveillancereports/20222023season/.

