# Supplementary material for: Cost-Effectiveness of the Use of Adjuvanted Quadrivalent Seasonal Influenza Vaccine in Older Adults in Ireland
Source: Vaccines (Basel). 2023 May 3;11(5):933. doi: 10.3390/vaccines11050933 (PMC10221880; doi:10.3390/vaccines11050933)
Supplement: Supplementary file 1 [file vaccines-11-00933-s001.zip › vaccines-2345220-supplementary.pdf]

Supplementary Table 1. Assumptions of probabilities of events per influenza case in low- and high-risk groups [23]

Age group	Probability of GP visits per case		Probability of hospitalization per case		Probability of deaths per hospitalization	
	Low risk	High risk	Low risk	High risk	Low risk	High risk
**6–23 months**	0.100	0.100	0.03	0.032	0.00043	0.01745
**2–17 years**	0.100	0.100	0.0016	0.0103	0.00074	0.02443
**18–49 years**	0.100	0.100	0.0019	0.018	0.00607	0.03997
**50–64 years**	0.100	0.100	0.0058	0.034	0.00607	0.03997
**65–74 years**	0.100	0.100	0.0312	0.0569	0.18529	0.42852
**≥75 years**	0.100	0.100	0.0315	0.0575	0.18529	0.42852

Supplementary Table 2. Vaccine effectiveness estimates by strain and age group [25]

	Strain		
**Age group (years)**	**A/H1N1**	**A/H3N2**	**B[a]**
6–23 months	69%	43%	69%
2–17 years	69%	43%	69%
18–49 years	73%	35%	54%
50–64 years	73%	35%	54%
65–74 years	61%	24%	53%
≥75 years	61%	24%	48%

[a]Estimates were the same for B/Yamagata and B/Victoria

Supplementary Figure 1. (a) Susceptible-Exposed-Infected-Recovered influenza transmission model structure and (b) probability tree of outcomes from symptomatic infected.

(a)

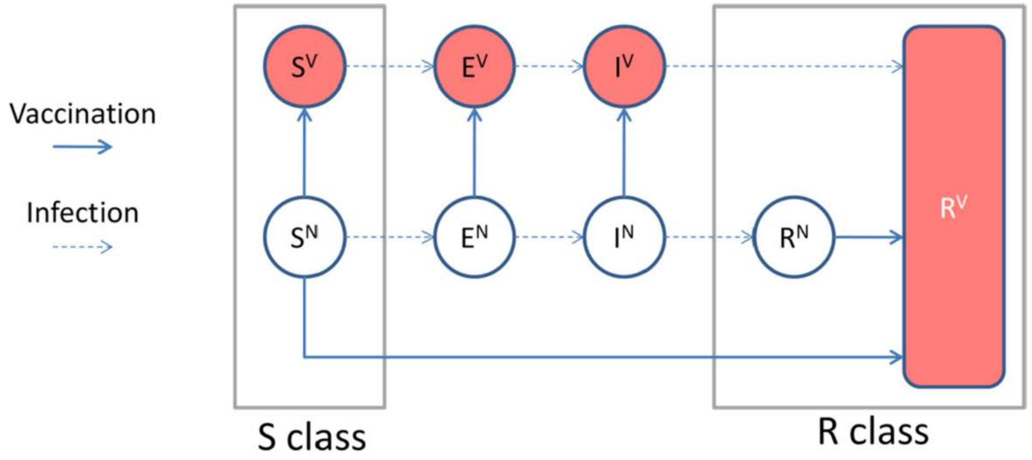

S^V, susceptible vaccinated; E^V, exposed vaccinated; I^V, infected vaccinated; R^V, recovered vaccinated; S^N, susceptible non-vaccinated; E^N, exposed non-vaccinated; I^N, infected non-vaccinated; R^N, recovered non-vaccinated

(b)

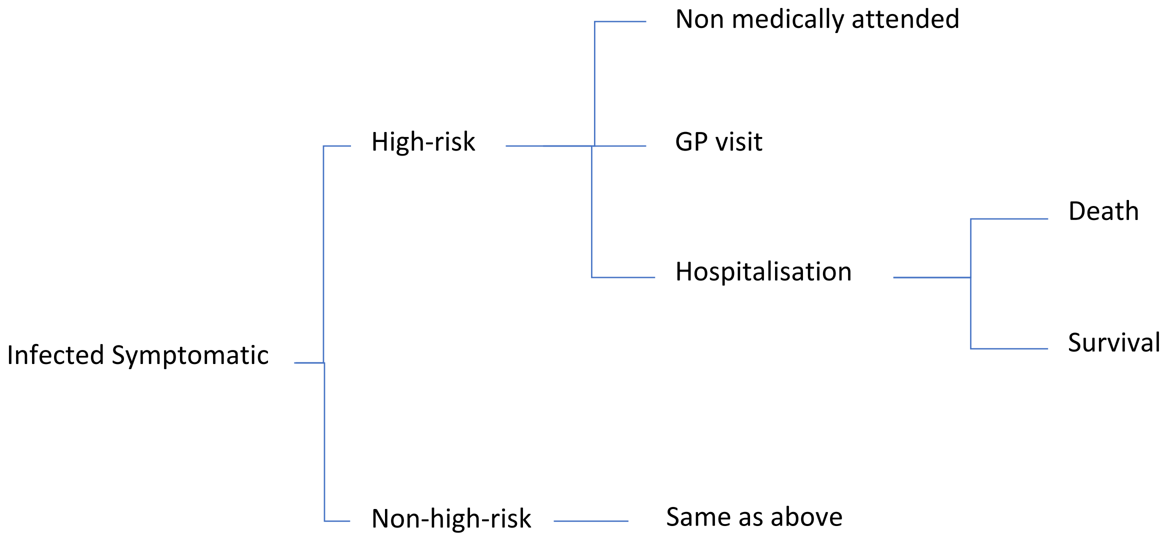

Non medically attended
High-risk
GP visit
Death
Hospitalisation
Infected Symptomatic
Survival
Non-high-risk
Same as above

Supplementary Figure 2: Model scenarios

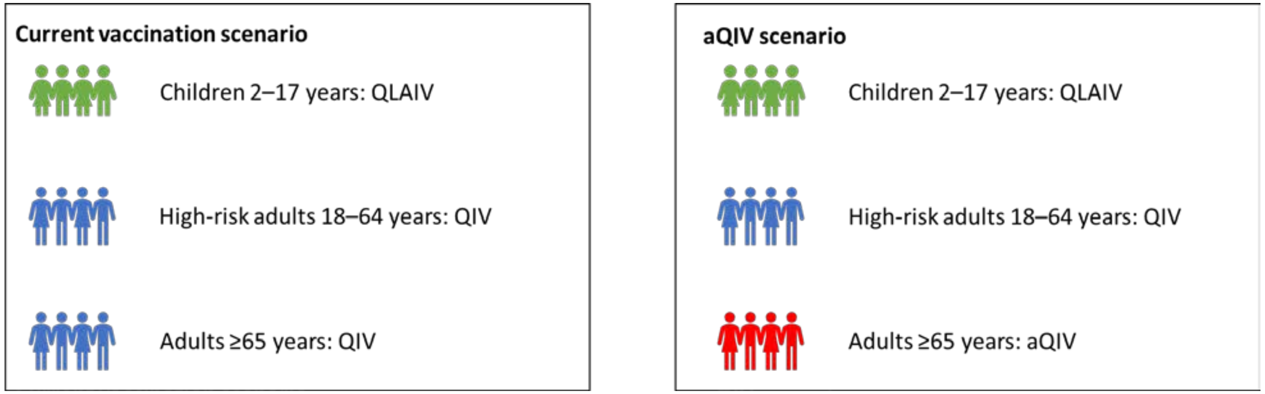

aQIV, adjuvanted quadrivalent influenza vaccine; QIV, quadrivalent influenza vaccine; QLAIV, quadrivalent live attenuated influenza vaccine
